# Application of an ultrasound-guided bilateral erector spinae plane block after the Nuss procedure for pectus excavatum in children: a retrospective cohort study with propensity score matching

**DOI:** 10.3389/fped.2023.1201604

**Published:** 2023-06-28

**Authors:** Shihuan Wu, Jing Wu, Xifeng Zhang

**Affiliations:** Department of Anesthesiology, Children's Hospital of Nanjing Medical University, Nanjing, China

**Keywords:** bilateral erector spinae plane block, funnel chest, Nuss procedure, thoracic epidural anesthesia, children

## Abstract

**Objective:**

To retrospectively analyze the effect of applying an ultrasound-guided bilateral erector spine plane block (ESPB) after the Nuss procedure for surgical repair of pectus excavatum (PE) in children.

**Methods:**

The subjects of the study were patients with severe PE who received the Nuss procedure in our hospital between 1 January 2019 and 30 November 2021. According to different methods for postoperative pain management, the enrolled patients were divided into two groups, the ultrasound-guided ESPB group and the thoracic epidural analgesia (TEA) group. The primary outcome of this study was analgesic drug dosage and the secondary outcome was numerical rating scales (NRSs) between the two groups.

**Results:**

There was no significant difference between the two groups in terms of demographic, preoperative clinical evaluation, or surgical characteristics (*P* > 0.05). The catheter duration in the TEA group was significantly shorter than that in the ESPB group (*P* < 0.05), while the hospitalization time in the ESPB group was significantly shorter than that in the TEA group (*P* < 0.05). In terms of oral morphine equivalent comparison, the required dose of the TEA group was lower than that of the ESPB group on the 1st and 2nd day after the operation (*P* < 0.05), and there was no statistical difference between the two groups on the 3rd and 4th day after the operation (*P* > 0.05). The number of patients with an S-NRS ≥ 7 and D-NRS ≥ 7 in the TEA group at day 1 was lower than that in the ESPB group (*P* < 0.05). There was no significant difference between the two groups at other time points (*P* > 0.05),

**Conclusion:**

An ultrasound-guided ESPB used in Nuss surgery for children with funnel chest can provide good analgesia for surgery and shorten the postoperative rehabilitation and hospitalization time of patients. It is a safe and effective alternative to TEA.

## 1. Introduction

Pectus excavatum (PE) is the most common congenital chest wall abnormality, with an incidence of 1/400 children (1). PE is more common in men than in women, with a ratio of 5:1 (2). It is characterized by anterior chest wall depression caused by a dorsal deviation of the sternum and the third to seventh ribs or costal cartilage (3). At present, the modified Ravitch procedure and the Nuss procedure are two commonly used surgical methods for the repair of PE (4, 5). The modified Ravitch surgery is highly invasive, causing great damage to the pectoralis major and rectus abdominis muscles of children, and involves the resection of malformed cartilage, resulting in higher postoperative pain scores and serious complications (6). The Nuss procedure is a minimally invasive thoracoscopic technique that does not require cartilage resection or osteotomy (7). However, after the placement of pectus bars for the surgical correction of the sternum, the costovertebral joint dislocation will also induce severe postoperative pain. The erector spine plane block (ESPB) has been used for postoperative pain control after thoracic and abdominal surgery, with a good local analgesic effect (8). Moreover, under ultrasound guidance, the catheter can be placed during surgery and used for the next few days (9). This study employed a new and safe ESPB with good pain relief effects after the Nuss procedure, which is reported as follows.

## 2. General data and methods

### 2.1. Research design and methods

A retrospective analysis was conducted on the clinical data of patients with severe PE who received the Nuss procedure in our hospital between 1 January 2019 and 30 November 2021. All patients underwent the Nuss procedure and postoperative pain management with ESPB or thoracic epidural analgesia (TEA). The patient data were as follows: clinical details (severity of the CT Haller index, preoperative symptom complaints, presence of scoliosis, normal and abnormal echocardiogram, ECG and/or pulmonary function test), postoperative analgesic drug dosage, numerical rating scale (NRS) results, and length of hospital stay (LOS) (10, 11). The general data of all patients showed comparability, and this study was reviewed and approved by the Medical Ethics Committee of our hospital (2022-KY008-015).

**Figure 1:**
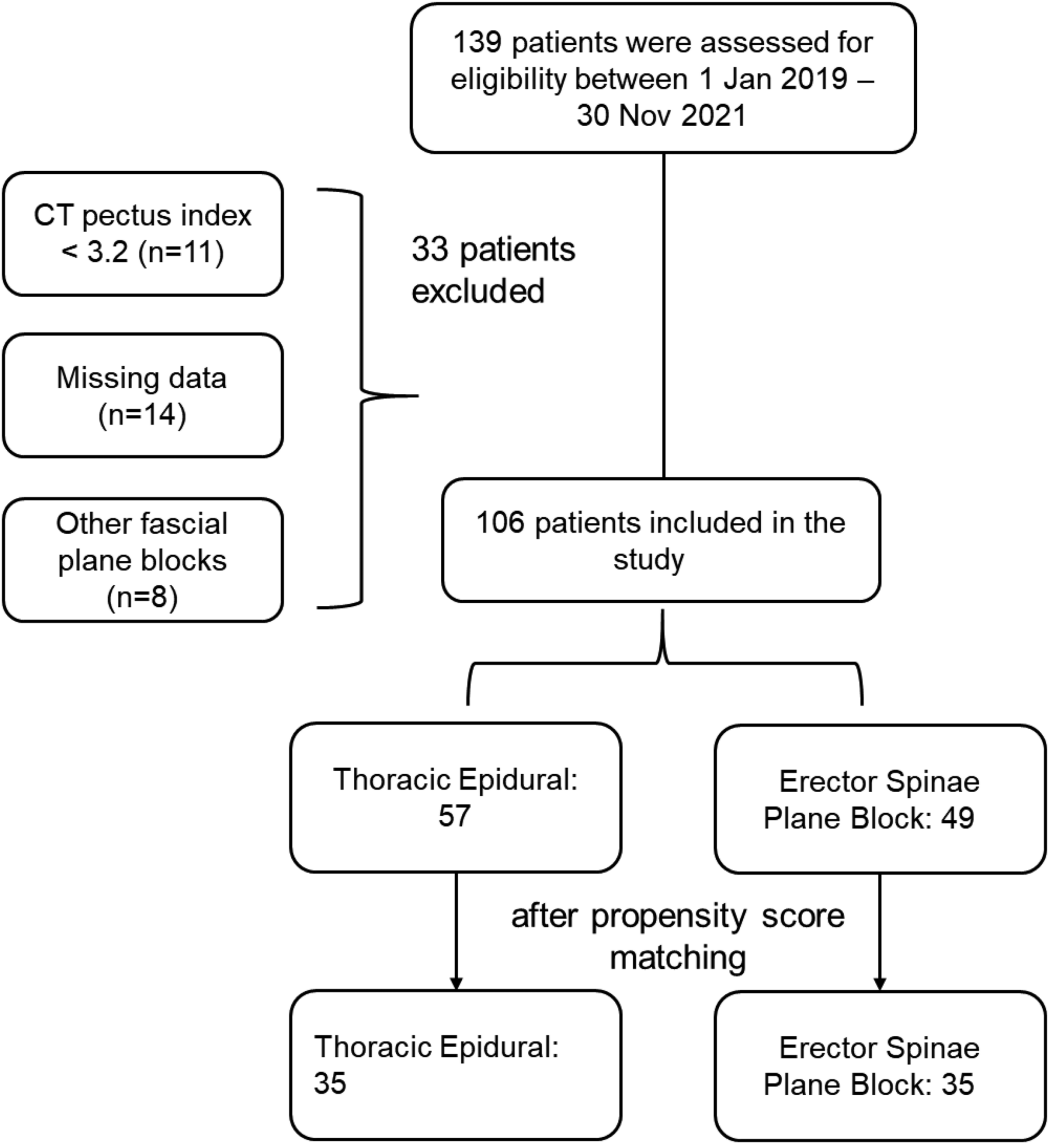
Flowchart to select the study population.

The inclusion criteria of eligible patients are described as follows: patients with a CT Haller index ≥3.2, worsened chest wall deformity over time, dyspnea with exercise intolerance, negative body image, uncommon results of echocardiogram, and an electrocardiogram and/or pulmonary function examination with an abnormal lung volume (Figure 1).

### 2.2. Operation methods

All children strictly abstained from food and water before surgery. After entering the room, venous access was established and the physiological indices of the children were routinely detected. Propofol and remifentanil were used for anesthesia induction.

#### 2.2.1. Erector pine plane block

During general anesthesia, the patients were provided with the placement of a bilateral single-injection ESPB catheter from head-to-toe under ultrasound guidance, and the tip of the catheter was located at the dermatomere level where there was the biggest depression in the sternum (usually T5–T6), and the level at which injection was performed was determined by a clear identification of the erector spinae muscle above the transverse process. Conduit tunneling was employed to minimize leakage. For each catheter, 15–20 mL 0.25% bupivacaine was generally used for infusion (total dose of <0.5 mg/kg), followed by an infusion of 0.2% ropivacaine from 6 mL/h (the maximum limit, 0.25 mg/kg/h/catheter). A continuous infusion of ropivacaine was performed for 5–7 days after the operation. Inpatients were infused via the CADD® electronic pump and continued to be infused with a portable pump device (ON-Q pump, Avanos Medical) after discharge until it was instructed to stop and withdraw. When the patients complained of pain (NRS < 7) or a body temperature >38.5°C, acetaminophen and ibuprofen were given alternately every 3 h. The patients were also given oxycodone (0.1 mg/kg or 5 mg at the maximum dose) when the NRS ≥ 7 and were orally administered diazepam for managing anxiety and/or relaxing the muscles.

#### 2.2.2. Thoracic epidural analgesia

The epidural catheter was placed in patients under general anesthesia, and the tip of the catheter was located in the skin with the largest defect. The catheter was used to infuse local anesthetics intraoperatively; then, 0.2% ropivacaine and 2–5 μg/mL hydromorphone were used as epidural analgesics, which were titrated to the basal rate of sufficient clinical effect. Ibuprofen and acetaminophen were used in combination with opioids or muscle relaxants to relieve penetrating pain similar to that of the ESPB group.

Acetaminophen and ibuprofen were administered alternately every 3 h, depending on the child's pain.

### 2.3. Data analysis

Oral opioid and doses: names of any oral and intravenous opioid administered, times for administration, and opioid doses were collected. The use of oral morphine equivalent (OME) was analyzed and compared between the two groups, including “total OME” and “OME per hour,” and the conversion rates of other anesthesia drugs and OME were calculated: Oxycodone 1:1.5 OME; Methadone 1:2 OME; Epidural Hydromorphone [where the total amount is the total daily volume (milliliters) multiplied by the hydromorphone concentration] 1:15 OME; IV Hydromorphone 1:15 OME; IV Morphine 1:3 OME; IV Fentanyl 100 (μg):20 (mg) OME.

The NRS was used to score on a scale of 0–10, with 0 representing no pain and 10 representing severe pain. Resting state NRS (S-NRS) and dynamic NRS (D-NRS) at light cough were assessed in all children.

SPSS 20.0 was used for all statistical analyses. The continuous variable data between the two groups were compared by using the independent sample *t*-test. The categorical variable data were expressed as percentage and tested by using the *χ*^2^ test. A score of *P* < 0.05 was considered statistically significant.

## 3. Results

### 3.1. Demographic and surgical characteristics of patients in the two groups

Before propensity score matching, significant differences were found in the parameters of age, body weight, and BMI between the two groups (*P *< 0.05), and baseline characteristics were not comparable. After matching propensity score, there was no statistically significant difference in demography between the two groups (*P *> 0.05), indicating comparability (Table 1). Before propensity score matching, there were differences in the total OR time and length of hospital stay between the two groups. After propensity score matching, the duration of analgesic catheterization in the TEA group was shorter than that in the ESPB group (*P *< 0.05), and the length of hospital stay in the ESPB group was significantly shorter than that in the TEA group (*P *< 0.05; Table 2).

**Table 1:** Demographic characteristics of patients.

Parameter	Before matching		*P*	After matching		*P*
	TEA (*n* = 57)	EPSB (*n* = 49)		TEA (*n* = 35)	EPSB (*n* = 35)	
Age (years)	11.8 (1.8)	9.5 (2.4)	0.003	11.2 (1.7)	10.2 (2.1)	0.197
Gender (male, *N*)	53 (93.0%)	43 (87.8%)	0.173	28 (80%)	32 (91.4%)	0.111
Patient weight (kg)	58.1 (14.3)	55.2 (10.6)	0.005	55.4 (12.3)	56.1 (9.7)	0.412
BMI	20.9 (2.3)	19.6 (2.0)	0.127	19.8 (2.6)	20.3 (2.9)	0.572
ASA			0.179			0.129
I	44 (77.2%)	39 (79.6%)		31 (88.6%)	33 (94.3%)	
II	13 (22.8%)	10 (20.4%)		4 (11.4%)	2 (5.7%)	
CT pectus index	8.3 (2.6)	14.9 (2.6)	0.001	4.5 (1.5)	20.3 (2.9)	<0.001
Scoliosis	13 (22.8%)	8 (16.3%)	0.139	3 (8.6%)	6 (17.1%)	0.163
Preop. ECHO			0.004			0.587
Normal	21 (36.8%)	13 (26.5)		15 (42.9%)	11 (31.4%)	
Abnormal	15 (26.3%)	8 (16.3%)		4 (11.4%)	6 (17.1%)	
Not obtained	21 (36.8%)	28 (57.1%)		16 (45.7%)	18 (51.4%)	
Preop. EKG			0.062			0.468
Normal	15 (26.3%)	20 (35.1%)		11 (31.4%)	12 (34.3%)	
Abnormal	13 (22.8%)	14 (28.6%)		6 (17.1%)	5 (14.3%)	
Preop. PFT			0.127			0.251
Normal	16 (28.1%)	13 (26.5%)		10 (28.6%)	9 (25.7%)	
Abnormal	18 (31.6%)	15 (30.1%)		9 (25.7%)	9 (25.7%)	
Not obtained	23 (40.4%)	21 (42.9%)		16 (45.7%)	17 (48.6%)	

Values presented are mean (SD) or *N* (%).

**Table 2:** Operative and postoperative data.

Parameter	Before matching		*P*	After matching		*P*
	TEA (*n* = 57)	ESPB (*n* = 49)		TEA (*n* = 35)	ESPB (*n* = 35)	
Total OR time (h)	3.1 (1.2)	1.9 (0.8)	<0.001	1.5 (0.3)	1.4 (0.5)	0.308
Pain catheter duration (days)	6.5 (1.8)	5.9 (2.1)	0.068	2.9 (0.8)	5.3 (1.5)	<0.001
Follow-up method			0.152			0.321
Readmission	14 (24.6%)	5 (10.2%)		3 (8.6%)	2 (5.7%)	
ED visit	14 (24.6%)	5 (10.2%)		4 (11.4%)	2 (5.7%)	
Phone call	29 (50.9%)	33 (67.3%)		28 (80%)	31 (88.6%)	
LOS (days)	7.4 (2.8)	5.3 (1.4)	<0.001	4.3 (1.1)	3.1 (0.8)	<0.001

Values presented are mean (SD) or *N* (%).

### 3.2. Comparison of NRS scores between the two groups

The number of patients with S-NRS ≥ 7 and D-NRS ≥ 7 in TEA at day 1 were less than those in ESPB group (*P* < 0.05), and there was no significant difference between the two groups at other time points (*P *< 0.05; Table 3).

**Table 3:** Comparison of NRS scores (≥7) between groups.

Parameter	TEA (*n* = 35), *n* (%)	ESPB (*n* = 35), *n* (%)	*P*
Severe pain for rest (S-NRS ≥ 7)			
Day 1	6 (17.1)	13 (37.1)	0.038
Day 2	5 (14.3)	9 (25.7)	0.119
Day 3	2 (5.7)	3 (8.6)	0.322
Day 4	1 (2.9)	2 (5.7)	0.380
Severe pain while coughing (D-NRS ≥ 7)			
Day 1	7 (20)	18 (51.4)	0.004
Day 2	6 (17.1)	10 (28.6)	0.12
Day 3	3 (8.5)	5 (14.4)	0.225
Day 4	2 (5.7)	3 (8.6)	0.322

### 3.3. Comparison of analgesic dose between the two groups

**Figure 2:**
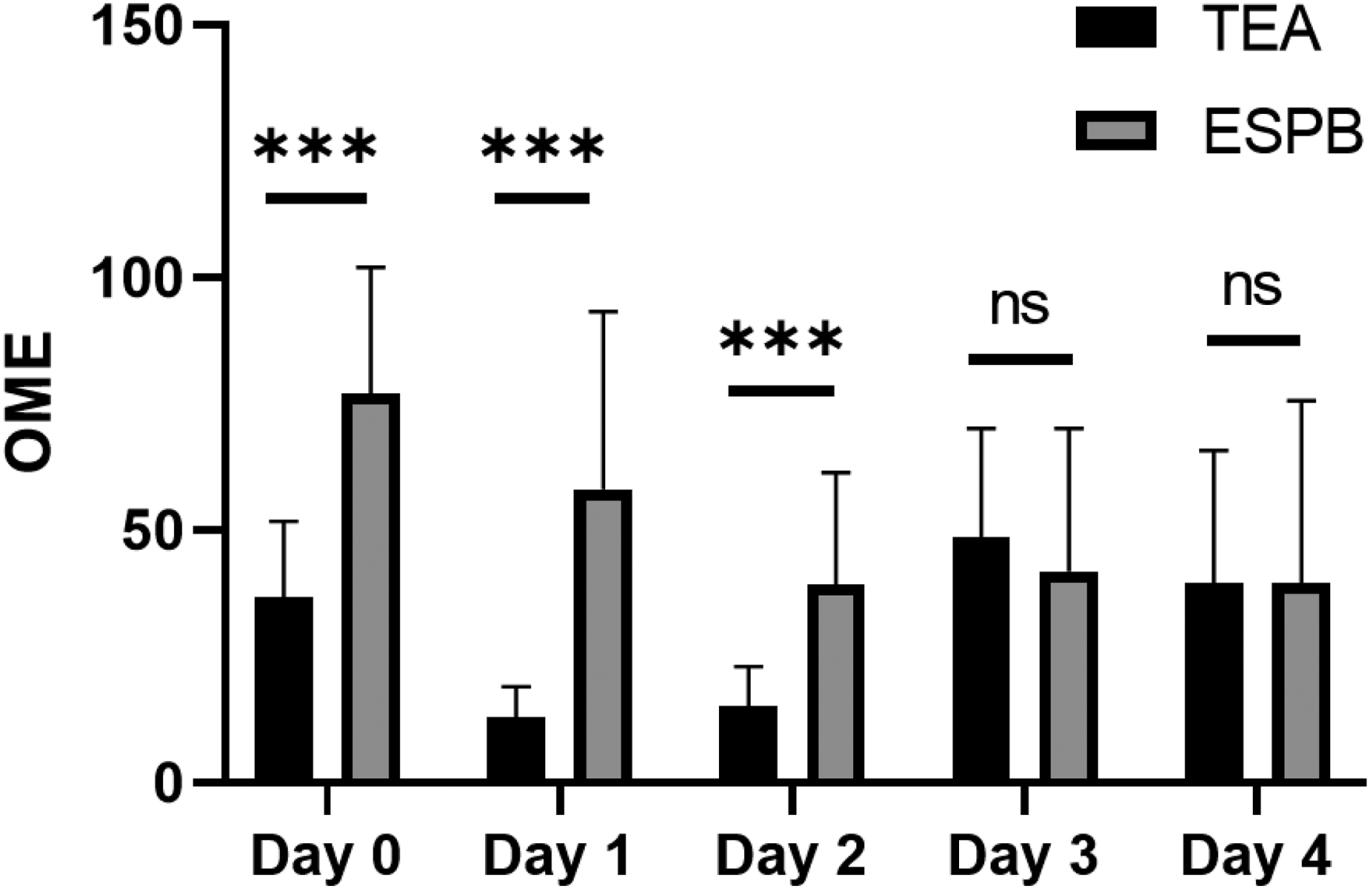
The results of OME of different times.

**Figure 3:**
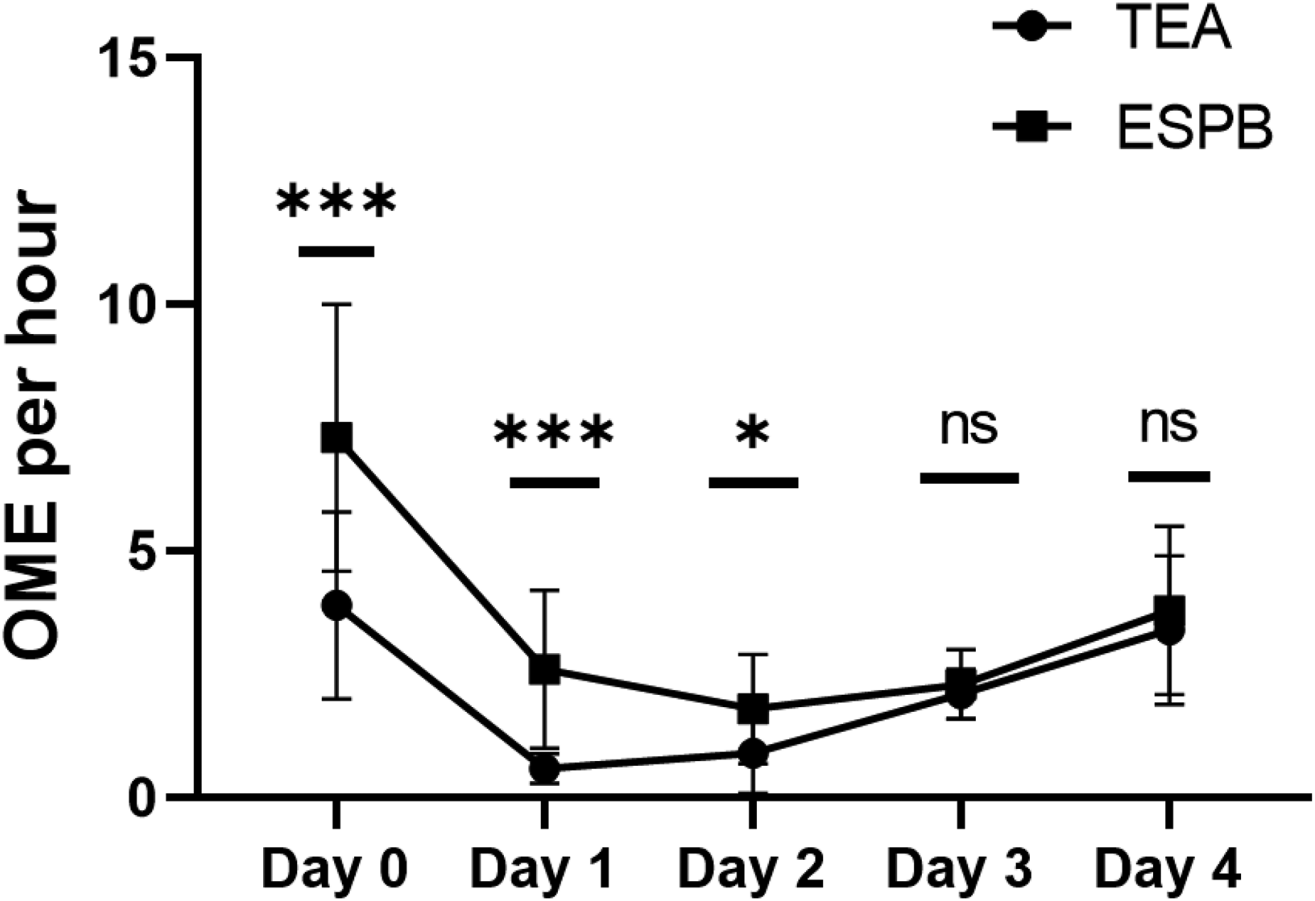
The results of OME per hour of different times. TEA, thoracic epidural analgesia; ESPB, erector spinae plane block.

On OME, the dose required by TEA was lower than that of the ESPB group on day 1 and 2 after surgery, and there was no difference between the two groups on day 3 and 4 after surgery (*P *< 0.05; Figures 2, 3).

## 4. Discussion

Postoperative analgesia is a difficult challenge in patients undergoing Nuss surgery for infundibular chest wall malformation, especially in the pediatric patient population (12). Analgesic therapy in children is largely single agent and intermittent, although very helpful when given (13). The pain experienced by children constantly requires special daily attention from health professionals aimed at mitigating the components of intractable pain and resolving those of treatable pain and to measure and implement strategies that prevent the dysfunction that causes pain (14). Therapeutic anesthesia generally includes patient-controlled intravenous analgesia, TEA, paravertebral block (PVB), a subcutaneous catheter for infusion, and cryoanalgesia (15–18). TEA or cryoanalgesia has been the primary option for postoperative analgesia in many treatment centers. However, TEA is suitable only for inpatients undergoing surgery, and is accompanied by a low risk of catastrophic nerve injury. Commonly, it requires the placement of the Foley catheter and may prolong the patients' LOS in the hospital due to the difficulty in transition from epidural analgesia (19, 20). Meanwhile, cryoanalgesia requires an additional port for placement, which may prolong the operation time and may cause nerve injury, leading to neuropathic pain (21).

The Nuss procedure has been recognized to be a minimally invasive thoracoscopic technique. Nevertheless, according to multiple studies, the perioperative pain of the Nuss procedure is equivalent to or even more severe than that of traditional surgery (22, 23). Although it is widely recognized that analgesia is of great significance and exercises great influence on the operation and postoperative rehabilitation of patients, there is controversy about the best technique and program of postoperative analgesia (24). TEA has been considered the gold standard for postoperative pain relief for many years. Owing to potential severe postoperative pain, TEA has been widely used to provide analgesia for patients undergoing repair surgery for PE (25). Despite good postoperative analgesia, TEA may still have risks of hypotension, infection, epidural hematoma, etc. According to a recent report, a less-invasive trunk block (e.g., PVB and ESPB) has been successfully applied for pain relief in this procedure (26). Nardiello and Herlitz showed that the application of ultrasound-guided ESPB in postoperative analgesia in children with pectinus excavatus can reduce the use of opioids and shorten the intensive care unit stay time (27). Currently, few studies have compared ESPB with standard intravenous analgesia in the analgesic treatment after PE surgery.

In our study, there was no significant difference in demographic, preoperative clinical evaluation, or surgical characteristics between the two groups (*P *> 0.05). In comparison with perioperative characteristics, the catheter duration in the TEA group was significantly shorter than that in the ESPB group (*P *< 0.05), while the length of hospital stay in the ESPB group was significantly shorter than that in the TEA group (*P *< 0.05). As for OME, the dose required in the TEA group was lower than that in the ESPB group on day 1 and day 2 after the operation, yet without any difference between the groups on day 3 and day 4 after the operation. The ESPB group did receive higher doses of opioids in the first 72 h after surgery, which could be due to several factors. The maximum anesthetic dose may be achieved by bilateral catheter infusion (6 mL/h) below the vertical spinal plane. Anatomical differences between the erector ridge plane and the epidural space may also lead to changes in the diffusion level of local anesthesia. Forer et al. reported that local anesthetics were diffused along the paravertebral region from the T5 erector ridge block, and the expansion of the diffusion area led to an increase in anesthetic use (28). In the same way, the diffused anesthetics provide good coverage of the Nuss operation and improve analgesia. In addition, epidural opioid delivery may differ in efficacy from systemic opioid delivery (29). The relatively shallow injection site reduces the risk of nerve damage, and patients have a lower risk of complications such as pneumothorax and hematoma. Compared with TEA, ESPB can achieve better analgesia in a shorter hospital stay and shorten the length of patient stay. Although patients occasionally complained of mild discomfort at the catheter outlet in the clinic, the catheter and covering dressing were generally well tolerated. After discharge, patients and their families can also easily remove these catheters at home, saving additional clinic visits. This study was limited to a single cohort center with a small sample size, and further multicohort studies are needed to present relevant conclusions.

Significantly, ESPB has several advantages. First, it avoids the risk of nerve injury caused by other methods of regional analgesia and can be applied in the outpatient department continuously as it has no relationship with hemodynamic fluctuation or respiratory damage. Second, compared with TEA, ESPB can help achieve excellent pain relief in a shorter LOS in the hospital. Third, it helps avoid the difficulty of transitioning from TEA to other forms of pain management. During TEA, there is a need for frequent and long-term use of the Foley catheter, which, however, can be withdrawn or completely avoided immediately after surgery. In clinical practice, patients occasionally complain of slight discomfort at the exit of the catheter. However, generally it is found that patients have good tolerance to the catheter and covering dressing. In addition, after discharge, patients and their families can voluntarily remove these catheters at home easily and conveniently, thus saving additional outpatient visits.

Finally, the limitations of this study may be related to the study design. As a retrospective cohort study, our research failed to provide reliable data on postoperative pain symptoms and/or pain medication use in outpatients. Consequently, it was only possible to measure the use of postoperative opioids during the postoperative period of inpatients in the TEA and ESPB groups. Further statistical analysis was invalid on the data of remaining inpatients. In addition, this was a single-center cohort study with a smaller sample size. Hence, the relevant findings in our research remain to be verified through a multicenter cohort study in the future.

In conclusion, ultrasound-guided ESPBs are a safe and effective alternative to TEA, which reduce pain after Nuss surgery in children with pectinus excavata and also reduce hospital stay for postoperative rehabilitation.

## Data Availability

The original contributions presented in the study are included in the article; further inquiries can be directed to the corresponding author.

## References

[1] Xu M Zhang G Gong J Yang J. Comparison of erector spinae plane and paravertebral nerve blocks for postoperative analgesia in children after the Nuss procedure: study protocol for a randomized controlled non-inferiority clinical trial. Trials. (2022) 23(1):139. 10.1186/s13063-022-06044-y 35164831 PMC8842927

[2] Fiorelli S Menna C Andreetti C Peritore V Rocco M Blasi RAD Bilateral ultrasound-guided erector spinae plane block for pectus excavatum surgery: a retrospective propensity-score study. J Cardiothorac Vasc Anesth. (2022) 36(12):4327–32. 10.1053/j.jvca.2022.08.018 36163156

[3] Fiorelli S Leopizzi G Saltelli G Andreetti C Fiorelli A Peritore V Bilateral ultrasound-guided erector spinae plane block for postoperative pain management in surgical repair of pectus excavatum via Ravitch technique. J Clin Anesth. (2019) 56:28–9. 10.1016/j.jclinane.2019.01.026 30684919

[4] Tulgar S Selvi O Senturk O Ermis MN Cubuk R Ozer Z. Clinical experiences of ultrasound-guided lumbar erector spinae plane block for hip joint and proximal femur surgeries. J Clin Anesth. (2018) 47:5–6. 10.1016/j.jclinane.2018.02.014 29522966

[5] Sertcakacilar G Kose S. Bilateral PECS II block is associated with decreased opioid consumption and reduced pain scores for up to 24 h after minimally invasive repair of pectus excavatum (Nuss procedure): a retrospective analysis. J Cardiothorac Vasc Anesth. (2022) 36(10):3833–40. 10.1053/j.jvca.2022.06.001 35817669

[6] Oh SK Lim BG Won YJ Lee DK Kim SS. Analgesic efficacy of erector spinae plane block in lumbar spine surgery: a systematic review and meta-analysis. J Clin Anesth. (2022) 78:110647. 10.1016/j.jclinane.2022.110647 35030493

[7] Bhushan S Huang X Su X Luo L Xiao Z. Ultrasound-guided erector spinae plane block for postoperative analgesia in patients after liver surgery: a systematic review and meta-analysis on randomized comparative studies. Int J Surg. (2022) 103:106689. 10.1016/j.ijsu.2022.106689 35662584

[8] Genc C Kaya C Bilgin S Dost B Ustun YB Koksal E. Pectoserratus plane block versus erector spinae plane block for postoperative opioid consumption and acute and chronic pain after breast cancer surgery: a randomized controlled trial. J Clin Anesth. (2022) 79:110691. 10.1016/j.jclinane.2022.110691 35220180

[9] Abu Elyazed MM Mostafa SF Abdelghany MS Eid GM. Ultrasound-guided erector spinae plane block in patients undergoing open epigastric hernia repair: a prospective randomized controlled study. Anesth Analg. (2019) 129(1):235–40. 10.1213/ANE.0000000000004071 30801359

[10] Macaire P Ho N Nguyen V Van HP Thien KDN Bringuier S Bilateral ultrasound-guided thoracic erector spinae plane blocks using a programmed intermittent bolus improve opioid-sparing postoperative analgesia in pediatric patients after open cardiac surgery: a randomized, double-blind, placebo-controlled trial. Reg Anesth Pain Med. (2020) 45(10):805–12. 10.1136/rapm-2020-101496 32817407

[11] Macaire P Ho N Nguyen T Nguyen B Vu V Quach C Ultrasound-guided continuous thoracic erector spinae plane block within an enhanced recovery program is associated with decreased opioid consumption and improved patient postoperative rehabilitation after open cardiac surgery—a patient-matched, controlled before-and-after study. J Cardiothorac Vasc Anesth. (2019) 33(6):1659–67. 10.1053/j.jvca.2018.11.021 30665850

[12] Marchetti G Vittori A Ferrari F Francia E Mascilini I Petrucci E Incidence of acute and chronic post-thoracotomy pain in pediatric patients. Children. (2021) 8(8):642. 10.3390/children8080642 34438533 PMC8392193

[13] Taylor EM Boyer K Campbell FA. Pain in hospitalized children: a prospective cross-sectional survey of pain prevalence, intensity, assessment and management in a Canadian pediatric teaching hospital. Pain Res Manag. (2008) 13(1):25–32. 10.1155/2008/478102 18301813 PMC2670807

[14] Marchetti G Vittori A Cascella M Mascilini I Piga S Petrucci E Pain prevalence and pain management in children and adolescents in an Italian third level pediatric hospital: a cross-sectional study. Ital J Pediatr. (2023) 49(1):41. 10.1186/s13052-023-01439-2 36978099 PMC10053721

[15] Bliss DP Jr Strandness TB Derderian SC Kaizer AM Partrick DA. Ultrasound-guided erector spinae plane block versus thoracic epidural analgesia: postoperative pain management after Nuss repair for pectus excavatum. J Pediatr Surg. (2022) 57(2):207–12. 10.1016/j.jpedsurg.2021.10.030 34949445

[16] Sertcakacilar G Pektas Y Yildiz GO Isgorucu O Kose S. Efficacy of ultrasound-guided erector spinae plane block versus paravertebral block for postoperative analgesia in single-port video-assisted thoracoscopic surgery: a retrospective study. Ann Palliat Med. (2022) 11(6):1981–9. 10.21037/apm-22-75 35400156

[17] Chen N Qiao Q Chen R Xu Q Zhang Y Tian Y. The effect of ultrasound-guided intercostal nerve block, single-injection erector spinae plane block and multiple-injection paravertebral block on postoperative analgesia in thoracoscopic surgery: a randomized, double-blinded, clinical trial. J Clin Anesth. (2020) 59:106–11. 10.1016/j.jclinane.2019.07.002 31330457

[18] Zhang Q Wu Y Ren F Zhang X Feng Y. Bilateral ultrasound-guided erector spinae plane block in patients undergoing lumbar spinal fusion: a randomized controlled trial. J Clin Anesth. (2021) 68:110090. 10.1016/j.jclinane.2020.110090 33096517

[19] Sifaki F Mantzoros I Koraki E Bagntasarian S Christidis P Theodoraki K. The effect of ultrasound-guided bilateral erector spinae plane block with and without dexmedetomidine on intraoperative and postoperative pain in laparoscopic cholecystectomies: a randomized, controlled, double-blind, prospective trial. Pain Physician. (2022) 25(7):E999–E1008. 36288585

[20] Kanna RM Ramachandran K Subramanian JB Shetty AP Rajasekaran S. Perioperative analgesic efficacy and safety of erector spinae plane block in posterior cervical spine surgery—a double blinded, randomized controlled study. Spine J. (2023) 23(1):6–13. 10.1016/j.spinee.2022.04.010 35470087

[21] van den Broek RJC Koopman JSHA Postema JMC Verberkmoes NJ Chin KJ Bouwman RA Continuous erector spinae plane block versus thoracic epidural analgesia in video-assisted thoracic surgery: a study protocol for a prospective randomized open label non-inferiority trial. Trials. (2021) 22(1):321. 10.1186/s13063-021-05275-9 33947442 PMC8094519

[22] Asar S Sari S Altinpulluk EY Turgut M. Efficacy of erector spinae plane block on postoperative pain in patients undergoing lumbar spine surgery. Eur Spine J. (2022) 31(1):197–204. 10.1007/s00586-021-07056-z 34802140

[23] Ciftci B Ekinci M Celik EC Yayik AM Aydin ME Ahiskalioglu A. Ultrasound-guided erector spinae plane block versus modified-thoracolumbar interfascial plane block for lumbar discectomy surgery: a randomized, controlled study. World Neurosurg. (2020) 144:e849–55. 10.1016/j.wneu.2020.09.077 32956890

[24] Piskin Ö Gökçe M Altinsoy B Baytar Ç Aydin BG Okyay RD Effects of continuous erector spinae plane block on postoperative pain in video-assisted thoracoscopic surgery: a randomized controlled study. Gen Thorac Cardiovasc Surg. (2022) 70(1):64–71. 10.1007/s11748-021-01687-1 34347237

[25] Kamel AAF Amin OAI Ibrahem MAM. Bilateral ultrasound-guided erector spinae plane block versus transversus abdominis plane block on postoperative analgesia after total abdominal hysterectomy. Pain Physician. (2020) 23(4):375–82. 10.36076/ppj.2020/23/375 32709172

[26] Xiong C Han C Zhao D Peng W Xu D Lan Z. Postoperative analgesic effects of paravertebral block versus erector spinae plane block for thoracic and breast surgery: a meta-analysis. PLoS One. (2021) 16(8):e0256611. 10.1371/journal.pone.0256611 34432822 PMC8386864

[27] Nardiello MA Herlitz M. Bilateral single shot erector spinae plane block for pectus excavatum and pectus carinatum surgery in 2 pediatric patients. Rev Esp Anestesiol Reanim. (2018) 65(9):530–3. 10.1016/j.redar.2018.04.006 29866441

[28] Forero M Adhikary SD Lopez H Tsui C Chin KJ. The erector spinae plane block: a novel analgesic technique in thoracic neuropathic pain. Reg Anesth Pain Med. (2016) 41(5):621–62. 10.1097/AAP.0000000000000451 27501016

[29] Zengin SU Ergun MO Gunal O. Effect of ultrasound-guided erector spinae plane block on postoperative pain and intraoperative opioid consumption in bariatric surgery. Obes Surg. (2021) 31(12):5176–82. 10.1007/s11695-021-05681-7 34449029

